# Serial Magnetic Resonance Imaging Leading to the Diagnosis of a Growing Skull Fracture in an Infant Without an Apparent Fracture Widening on Follow-Up Computed Tomography

**DOI:** 10.7759/cureus.114130

**Published:** 2026-08-07

**Authors:** Haruka Takemoto, So Kobayashi, Miho Sasaki, Masataka Inoue, Masato Ito, Hirokazu Arai

**Affiliations:** 1 Department of Pediatrics, Yuri Kumiai General Hospital, Yurihonjo, JPN; 2 Department of Pediatrics, Akita Red Cross Hospital, Akita, JPN; 3 Department of Neonatology, Akita Red Cross Hospital, Akita, JPN; 4 Department of Pediatrics, Akita University Graduate School of Medicine, Akita, JPN

**Keywords:** growing skull fracture (gsf), head trauma, infant, magnetic resonance imaging, skull fracture

## Abstract

A growing skull fracture (GSF), an infrequent complication of skull fractures in infancy and early childhood, is characterized by progressive fracture diastasis, dural defect, and meningeal and/or brain parenchymal herniation. We report the case of a three-month-old girl who developed GSF following head trauma. She was brought to our hospital because of decreased responsiveness after falling down the stairs while being carried by her mother. Initial head computed tomography (CT) revealed a right temporoparietal skull fracture with diastasis measuring 7-8 mm, acute subdural hematoma, traumatic subarachnoid hemorrhage, intraventricular hemorrhage, and bilateral parietal contusions. The acute phase was conservatively managed without clinical deterioration. On hospital day 8, magnetic resonance imaging (MRI) suggested dural injury and brain herniation at the fracture site. Although follow-up CT on day 27 post-injury demonstrated no apparent fracture widening, MRI on day 48 revealed clear dural disruption and cyst formation, resulting in the diagnosis of GSF. Cranioplasty was performed 75 days post-injury, and the postoperative course was uneventful. This case suggests that reliance on CT findings alone may delay GSF diagnosis in selected high-risk infants. In such patients, early and repeat MRI, timed according to the clinical and radiological course, may assist in detecting dural disruption, brain herniation, or cyst formation before obvious fracture progression becomes apparent on CT. The imaging intervals used in this patient should be interpreted as case-informed rather than as a validated surveillance protocol.

## Introduction

A growing skull fracture (GSF) is an uncommon, late-onset complication of skull fractures in infants and young children. It is characterized by progressive widening of the fracture, dural defect, and meningeal and/or brain parenchymal herniation [1,2]. Pathogenesis encompasses fracture diastasis accompanied by a dural tear, with subsequent progression attributable to brain growth and cerebrospinal fluid pulsation [3,4].

Early diagnosis and prompt surgical intervention are crucial for preventing progressive brain injury. However, in the early phase, fracture enlargement may not be apparent, causing variability in the timing of diagnosis [5,6]. Although computed tomography (CT) is valuable for evaluating fracture diastasis and bone defects, magnetic resonance imaging (MRI) is superior for detecting dural tears, brain herniation, and other findings suggestive of GSF progression [5-7].

We report an infant with GSF diagnosed using serial MRI during conservative management following head trauma. In this case, CT did not reveal apparent fracture progression, whereas MRI significantly contributed to diagnosis and treatment decision-making. The principal educational objective of this case is to highlight the potential role of risk-based serial MRI in high-risk infants when CT findings remain inconclusive.

## Case presentation

The patient was a three-month-old girl. She was born at 39 weeks and 4 days of gestation via vaginal delivery, with a birth weight of 3,498 g. Her Apgar scores were both 9 at 1 and 5 min. She had no significant medical history, and routine infant checkups detected no abnormalities.

On the day of injury, she fell from the third step of a staircase while being carried by her mother, striking her right temporal region. She was initially evaluated at a local pediatric clinic due to decreased responsiveness and was subsequently transferred to our hospital.

On admission, her weight, temperature, oxygen saturation, and heart rate were 6.5 kg, 36.4 °C, 94%, and 220 beats/min, respectively. Her Pediatric Glasgow Coma Scale score was 9 (E3V2M4). A 12 × 8.5-cm swelling was noted over the right temporal region. Her pupils measured 2 mm bilaterally with preserved light reflexes. Diminished left-sided responsiveness was noted; however, no seizures were observed. No other traumatic findings were identified.

Laboratory findings on admission are summarized in Table 1. The main abnormalities were leukocytosis and elevated levels of D-dimer and fibrin degradation products. Venous blood gas analysis showed hyperlactatemia and mild metabolic acidosis; other findings were not clinically remarkable. Chest and abdominal radiographs were unremarkable.

**Table 1:** Laboratory findings on admission Note: Reference values are representative pediatric ranges for a three-month-old infant and may vary by laboratory and assay.

Laboratory parameter	Result	Reference value
Complete blood count		
White blood cell count	18,300/μL	6,000-17,500/μL
Hemoglobin	11.8 g/dL	9.5-14.1 g/dL
Platelet count	639,000/μL	190,000-660,000/μL
Biochemistry		
Total bilirubin	0.31 mg/dL	<1.0 mg/dL
Alanine aminotransferase	49 U/L	7-55 U/L
Aspartate aminotransferase	40 U/L	15-58 U/L
Alkaline phosphatase	318 U/L	124-341 U/L
Gamma-glutamyl transferase	23 U/L	8-90 U/L
Lactate dehydrogenase	365 U/L	190-420 U/L
Creatine kinase	333 U/L	26-192 U/L
Blood urea nitrogen	8.2 mg/dL	2-20 mg/dL
Creatinine	0.23 mg/dL	0.2-0.4 mg/dL
Sodium	139 mmol/L	135-145 mmol/L
Potassium	5.3 mmol/L	3.4-5.6 mmol/L
Chloride	107 mmol/L	96-110 mmol/L
Calcium	10.5 mg/dL	8.9-10.5 mg/dL
Inorganic phosphorus	5.8 mg/dL	4.2-9.0 mg/dL
Total protein	6.0 g/dL	4.4-6.6 g/dL
Albumin	4.3 g/dL	3.8-5.4 g/dL
C-reactive protein	0.02 mg/dL	<0.5 mg/dL
Coagulation		
Prothrombin time	13.4 seconds	10.6-14.9 seconds
Activated partial thromboplastin time	31.1 seconds	26.5-52.5 seconds
Fibrinogen	195 mg/dL	150-362 mg/dL
D-dimer	15.5 μg/mL	0.11-0.42 μg/mL
Fibrin degradation products	28 μg/mL	<10 μg/mL
Venous blood gas analysis		
Venous pH	7.35	7.33-7.43
Venous partial pressure of carbon dioxide	31.1 mmHg	32-45 mmHg
Glucose	107 mg/dL	65-139 mg/dL
Bicarbonate	17 mmol/L	18-27 mmol/L
Base excess	-7.9 mmol/L	-3 to +3 mmol/L
Lactate	5.2 mmol/L	0.5-2.2 mmol/L

**Figure 1:**
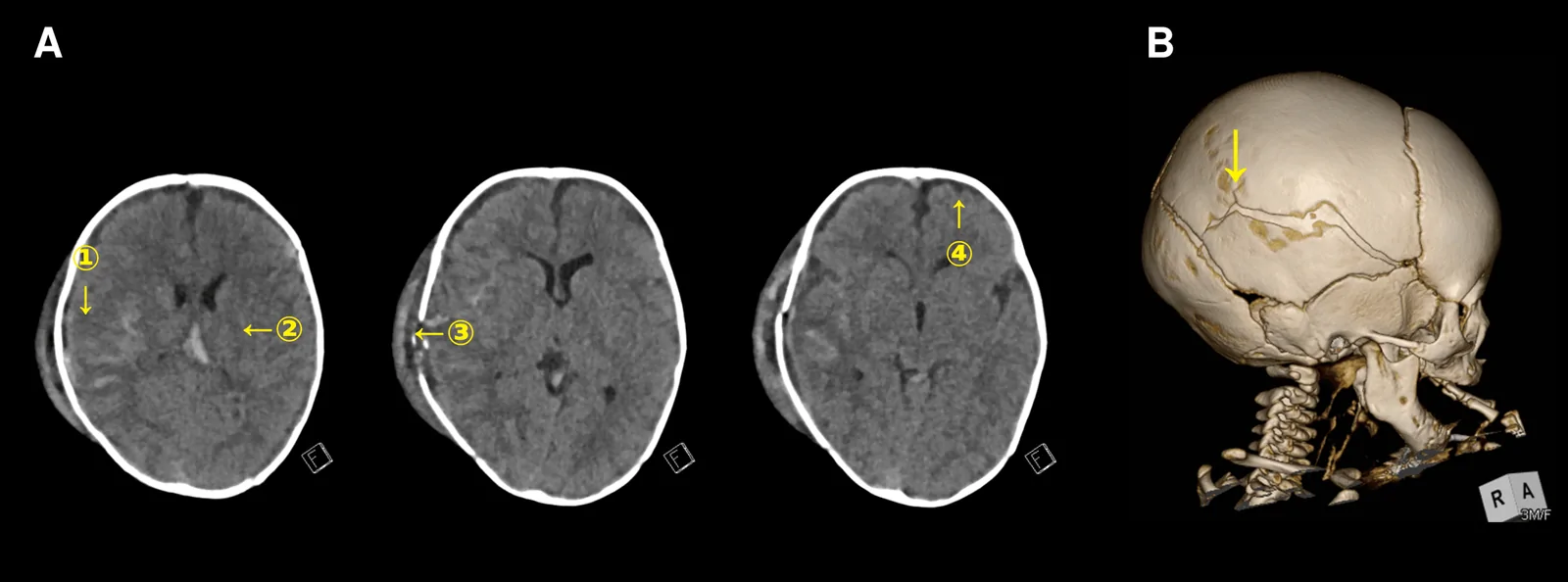
Initial head computed tomography (CT) obtained at 1 h post-injury (A) Axial CT images revealing associated traumatic intracranial injuries, including (1) right temporoparietal contusion with traumatic subarachnoid hemorrhage, (2) intraventricular hemorrhage, (3) acute subdural hematoma in the right temporal region, and (4) left temporoparietal contusion. (B) Three-dimensional reconstructed CT image demonstrating a right temporoparietal skull fracture with diastatic separation (arrow).

Head CT performed at approximately 1 h post-injury revealed a right temporoparietal skull fracture with diastasis measuring 7-8 mm. Furthermore, bilateral parietal contusions, traumatic subarachnoid hemorrhage, intraventricular hemorrhage, and acute subdural hematoma in the right temporal region were identified (Figure 1).

Conservative management, including fasting, intravenous fluids, head elevation, administration of a hemostatic agent, and levetiracetam, was initiated. Repeat CT at 2 and 6 h post-injury demonstrated no progression of the hematoma or scalp swelling. Follow-up CT on hospital days 2 and 5 also showed no worsening. By hospital day 7, the left-sided responsiveness had improved, and no new neurological deficits or seizures were observed. Levetiracetam was therefore discontinued on hospital day 7 and was not restarted at discharge because the patient remained seizure-free and neurologically stable.

**Figure 2:**
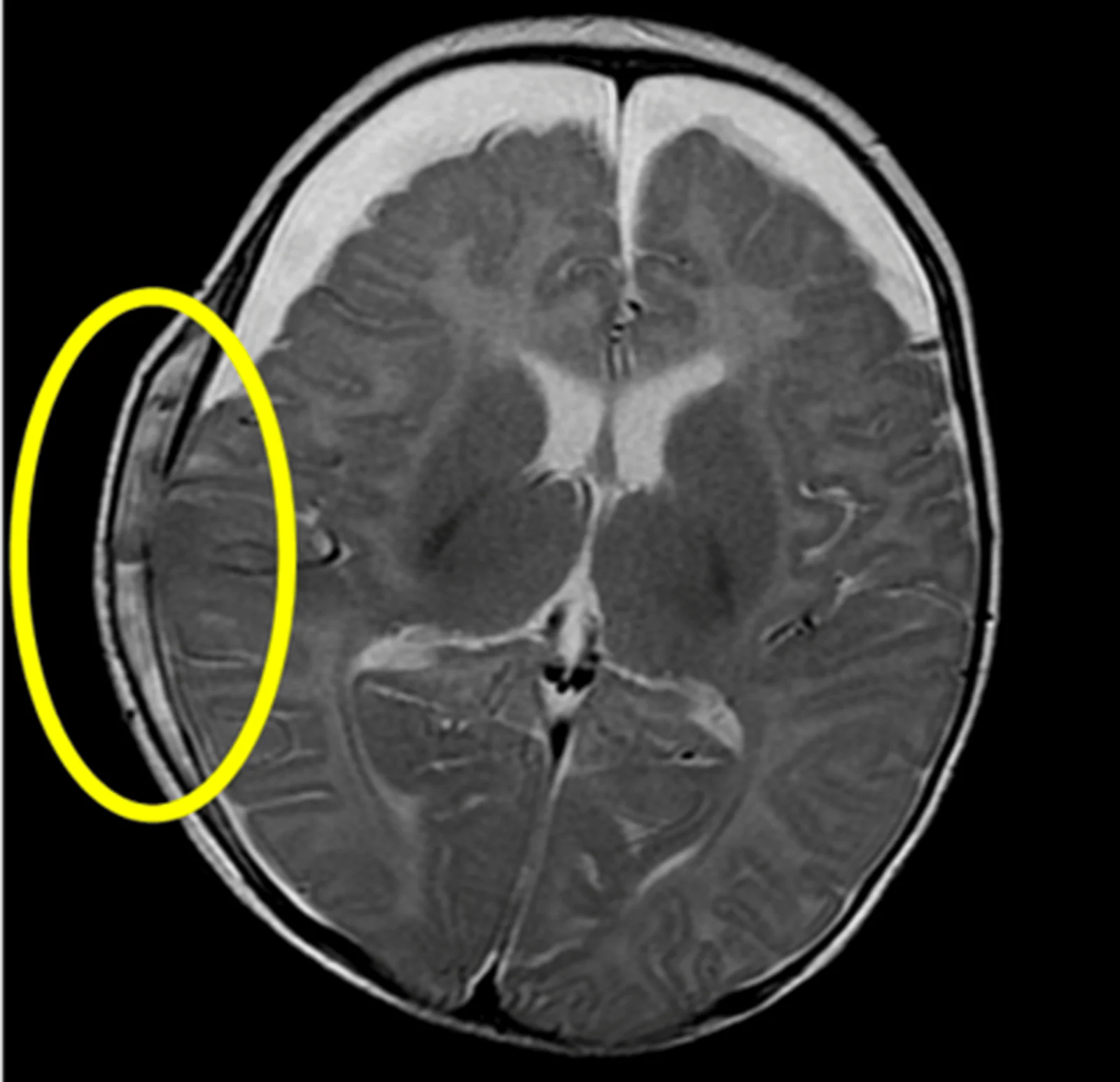
Axial T2-weighted magnetic resonance imaging obtained under sedation on hospital day 8 Suspected focal dural disruption and early brain herniation at the fracture site without a well-defined cystic lesion (circle). These findings were considered suggestive but not definitive for a growing skull fracture.

On hospital day 8, brain MRI was performed under sedation. The axial T2-weighted images presented in this report showed suspected focal dural disruption and early brain herniation at the fracture site; however, no well-defined cystic lesion was present. These findings were therefore considered suggestive but not definitive for GSF (Figure 2). We subsequently consulted neurosurgeons at a tertiary center. Because the patient remained neurologically stable, outpatient neurosurgical follow-up and planned imaging surveillance were continued. As part of the assessment of the injury mechanism, including possible non-accidental injury or abusive head trauma, an ophthalmologic examination was performed and revealed no vitreous hemorrhage. The patient was discharged in stable condition on hospital day 15.

**Figure 3:**
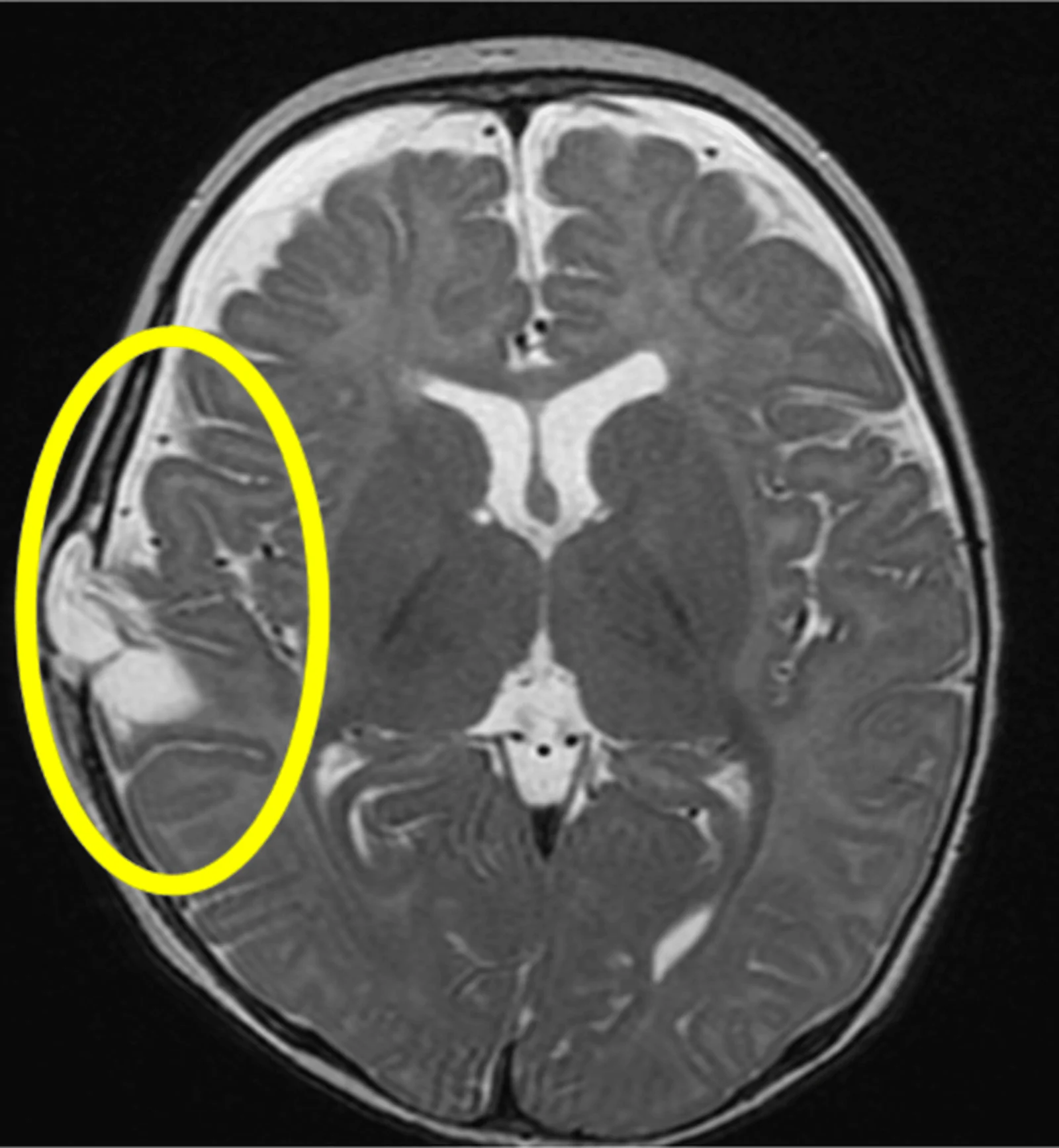
Axial T2-weighted magnetic resonance imaging obtained under sedation on day 48 post-injury Clear dural discontinuity accompanied by a newly developed cystic lesion and brain herniation at the fracture site (circle), supporting the definitive diagnosis of a growing skull fracture.

She was followed up as an outpatient. On day 27 post-injury, CT revealed no hematoma progression or apparent fracture widening. Repeat MRI on day 48 was also performed under sedation. The axial T2-weighted images presented in this report demonstrated clear dural discontinuity accompanied by a newly developed cystic lesion and brain herniation at the same site, leading to the definitive diagnosis of GSF (Figure 3). She was then referred to the tertiary neurosurgical center for operative management. Because she remained neurologically stable without progressive symptoms, elective surgical repair was scheduled following neurosurgical assessment.

Cranioplasty was performed 75 days following injury. Intraoperatively, a cystic lesion with fluctuation was noted beneath the periosteum. The herniated brain tissue was spontaneously reduced following incision and decompression. The dural defect was exposed circumferentially and repaired using a free galeal graft harvested from the subcutaneous tissue beneath the surgical field. The postoperative course was uneventful, and the patient was discharged eight days postoperatively.

**Figure 4:**
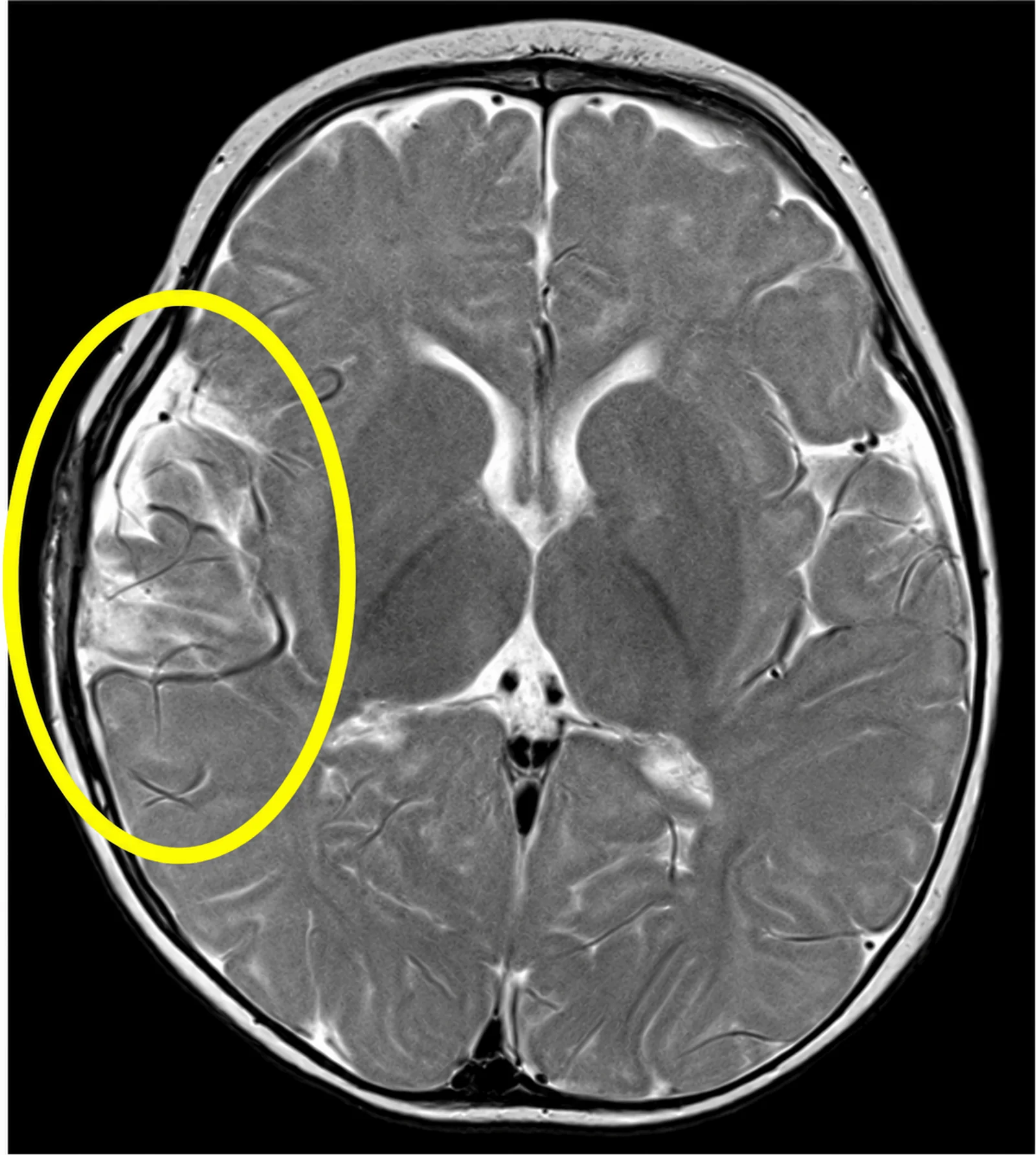
Postoperative MRI Reduction of the cystic lesion (circle) without hydrocephalus or progressive abnormalities.

MRI on day 119 following injury showed cystic lesion reduction (Figure 4). Follow-up MRI at one and two years postoperatively revealed no hydrocephalus or progressive lesions. To date, no apparent developmental delay or neurological sequelae have been observed.

## Discussion

GSF, an infrequent complication of skull fractures in infants and young children, is characterized by progressive fracture diastasis and herniation of intracranial contents through a dural defect [1,2]. A skull fracture accompanied by a dural tear provides the pathological basis for its development, with subsequent progression attributed to brain growth and cerebrospinal fluid pulsation [3,4].

In this case, multiple high-risk features of GSF progression were present from an early stage, including very young age, significant scalp swelling, intracranial injury with contusions, and a widely diastatic skull fracture measuring 7-8 mm. Previous studies have identified young age, scalp hematoma, fracture diastasis, underlying brain injury, and subdural fluid collection as risk factors for GSF [5,8,9]. A recent retrospective study of 190 infants with skull fractures found greater fracture splay and elevation or depression among the three patients who developed GSF and proposed focusing follow-up on fractures with displacement greater than 4 mm and/or elevation or depression greater than 3 mm [10]. Although the small number of GSF events limits generalizability, these findings support classifying the present patient as high risk and justify cautious imaging follow-up.

From a diagnostic standpoint, CT is valuable for evaluating fracture diastasis and bone defects, whereas MRI is more sensitive for detecting dural injury, brain herniation, and cyst formation [5,7,9]. In this case, MRI on hospital day 8 suggested focal dural disruption and early brain herniation, whereas CT on day 27 showed no apparent fracture widening. MRI on day 48 subsequently demonstrated clear dural discontinuity and cyst formation, leading to the definitive diagnosis of GSF. This clinical course suggests that MRI may detect GSF-related soft-tissue progression even when apparent osseous progression is absent on CT. Dural disruption, cerebrospinal fluid pulsation, early cyst formation, and brain herniation may precede sufficient bone resorption or remodeling to produce visible fracture widening on CT.

Routine MRI for all infants with skull fractures may not be feasible because of limitations in availability, the need for sedation, cost, and institutional practice. Therefore, a selective, risk-based approach may be more practical. High-risk features include very young age, significant scalp swelling or cephalhematoma, fracture diastasis of 4 mm or greater, underlying brain injury, and imaging findings suggestive of dural disruption or brain herniation [5,8-11]. Recent literature also illustrates variability in the timing of GSF progression. One case developed clear GSF progression within three weeks of injury [12], while recent technical guidance recommends MRI assessment after displaced fractures and clinical follow-up for simple skull fractures [13]. These observations support individualized surveillance rather than a fixed imaging schedule.

When initial MRI findings are suggestive but inconclusive, and the patient remains clinically stable, planned repeat MRI may be considered according to the clinical and radiological course. In this patient, MRI on hospital day 8 raised suspicion for GSF, whereas CT on day 27 showed no apparent fracture widening. Repeat MRI on day 48 demonstrated definite dural disruption and cyst formation, prompting renewed neurosurgical assessment and subsequent repair. This period of reassessment is broadly consistent with recommendations that young children with skull fractures undergo clinical follow-up during the first one to two months to assess for GSF [14]. However, this report describes a single patient and cannot establish the optimal timing or frequency of MRI surveillance. The imaging intervals of approximately one to two weeks and four to six weeks reflect this patient’s clinical course and should be regarded as a case-informed, risk-based approach rather than a validated surveillance protocol. Imaging decisions should be individualized according to clinical progression, fracture characteristics, MRI availability, need for sedation, cost, institutional practice, and neurosurgical assessment. Progressive scalp swelling, a palpable skull defect, seizures, or new neurological symptoms should prompt expedited imaging.

The principle of GSF treatment is to halt disease progression by closing the dural defect and performing cranioplasty when needed [4,15]. Recent literature continues to emphasize meticulous dural closure and appropriate reconstruction of the bony defect [13]. A contemporary pediatric neurosurgical series also reported favorable outcomes with early intervention and the use of autologous grafts for duraplasty and cranioplasty [16]. In the present case, surgical repair was performed 75 days post-injury, resulting in reduction of the cystic lesion and a favorable long-term outcome without hydrocephalus or apparent neurological sequelae. This course supports the clinical importance of prompt recognition, scheduled reassessment, and timely neurosurgical intervention in selected high-risk infants [4,5,13,15,16].

## Conclusions

We report an infant with GSF diagnosed using serial MRI during conservative management and subsequently treated surgically. This case suggests that follow-up of high-risk skull fractures should not rely solely on CT evidence of fracture widening. In selected high-risk infants with inconclusive CT findings, early and repeat MRI may be considered according to the clinical and radiological course. The specific imaging intervals used in this patient should be regarded as case-informed rather than as an established surveillance protocol and require validation in larger studies. Close neurosurgical collaboration may facilitate timely diagnosis and intervention before progressive neurological damage occurs.

## References

[1] LE RA, ER TC (1961). Growing skull fractures of childhood. J Neurosurg.

[2] Choi JI, Kim SD (2022). Pediatric minor traumatic brain injury: growing skull fracture, traumatic cerebrospinal fluid leakage, concussion. J Korean Neurosurg Soc.

[3] Scarfò GB, Mariottini A, Tomaccini D, Palma L (1989). Growing skull fractures: progressive evolution of brain damage and effectiveness of surgical treatment. Childs Nerv Syst.

[4] Muhonen MG, Piper JG, Menezes AH (1995). Pathogenesis and treatment of growing skull fractures. Surg Neurol.

[5] Wang X, Li G, Li Q, You C (2013). Early diagnosis and treatment of growing skull fracture. Neurol India.

[6] Ersahin Y, Gülmen V, Palali I, Mutluer S (2000). Growing skull fractures (craniocerebral erosion). Neurosurg Rev.

[7] Husson B, Pariente D, Tammam S, Zerah M (1996). The value of MRI in the early diagnosis of growing skull fracture. Pediatr Radiol.

[8] Zhao Q, Ying J, Chen Y, Chen F, Zhang T, Jing J (2024). Clinical and imaging characteristics of growing skull fractures in children. Sci Rep.

[9] Singh I, Rohilla S, Siddiqui SA, Kumar P (2016). Growing skull fractures: guidelines for early diagnosis and surgical management. Childs Nerv Syst.

[10] John W, Lowes D, Leach P (2026). Follow up of infants with skull fractures by neurosurgeons due to the risk of growing fractures; is it needed?. Br J Neurosurg.

[11] Matsuura H, Omama S, Yoshida Y (2012). Use of magnetic resonance imaging to identify the edge of a dural tear in an infant with growing skull fracture: a case study. Childs Nerv Syst.

[12] Park WH, Kim SJ, Yun JH (2024). Successful treatment of growing skull fracture that developed only 3 weeks after injury: a case report. Korean J Neurotrauma.

[13] Etter MM, Vernik T, Licci M, Guzman R, Soleman J, Greuter L (2025). Surgical management of growing skull fractures: how I do it. Acta Neurochir (Wien).

[14] Babl FE, Tavender E, Ballard DW (2021). Australian and New Zealand guideline for mild to moderate head injuries in children. Emerg Med Australas.

[15] Liu XS, You C, Lu M, Liu JG (2012). Growing skull fracture stages and treatment strategy. J Neurosurg Pediatr.

[16] Tahir MZ, Mirza FA, Thompson DN, Hayward R (2024). Early intervention and use of autologous grafts in growing skull fractures results in better outcomes: experience from a tertiary pediatric neurosurgery center. Oper Neurosurg.

